# The overlap phenotype: the (missing) link between asthma and COPD

**DOI:** 10.1186/2049-6958-7-8

**Published:** 2012-06-20

**Authors:** Barbara Piras, Marc Miravitlles

**Affiliations:** 1 Institute of Respiratory Diseases and TB, University of Sassari, 21 Università Square, 07100, Sassari, Italy; 2 Institut d’Investigacions Biomèdiques August Pi i Sunyer (IDIBAPS), Hospital Clínic, Ciber de Enfermedades Respiratorias (CIBERES), Villarroel 170, 08036, Barcelona, Spain

## 

The definition of chronic obstructive pulmonary disease (COPD) as a preventable and treatable condition characterized by a not completely reversible chronic airflow obstruction [1] is so broad and imprecise that many different types of patients with distinct clinical characteristics, prognosis and response to treatments may fit in. These different types are now described as “clinical phenotypes” and the interest in their definition and characterisation is growing [2]. Among these phenotypes, the so-called overlap of syndromes with airflow obstruction is usually poorly considered [3].

In a first step of phenotyping, a COPD patient can potentially be classified as a predominant parenchymal destructive or predominant airflow limitation phenotype by using a few clinical, radiological and functional findings [4]. However, when a patient presents characteristics and symptoms of two or more respiratory diseases at the same time, this is described as an overlap syndrome. In particular, the asthma-COPD overlap phenotype has been described as symptoms of increased variability of airflow in association with an incompletely reversible airflow obstruction [5]. From a clinical point of view it usually corresponds to individuals diagnosed with asthma before the age of 40 who, at an older age, fulfil the criteria for COPD [6]. Recent estimations of its prevalence report that about 13-20% of subjects with COPD have the overlap phenotype [6,7], with an increasing trend in the elderly population (up to 50% in those aged over 70 years) [7]. Since they have been systematically excluded from both COPD and asthma pharmacological clinical trials as not being “pure” subjects, it is clear that we cannot really know the response to pharmacological treatment of a significant number of patients with COPD.

Increased reversibility is one of the key differential aspects of individuals with the overlap asthma-COPD phenotype. Reversibility in COPD is not only possible but it is also not so infrequent: indeed, significant reversibility in COPD is observed frequently in everyday clinical practice and in a series of patients included in the newly designed clinical trials [8]. Over one half to almost two-thirds of the patients with moderate-to-very-severe COPD participating in the large clinical trial UPLIFT met the most commonly used criteria for acute bronchodilator responsiveness [9]. In a recent study of COPD patients without a prior history of asthma, 44% of the subjects showed a positive bronchodilator test with an inverse correlation between bronchodilator reversibility and the GOLD severity stages [10].

Reversibility, however, has not been considered to be a reliable parameter to diagnose or classify patients; the reason being that a patient may be reversible or irreversible in different determinations at different time points [11]. This is a consequence of dichotomising a continuous variable as positive or negative. In fact, this approach could be valid for epidemiologic studies but should never be used to guide clinical decisions in an individual patient. Reversibility in clinical practice must be considered as a continuous variable, and therapeutic decisions should be made according to the magnitude of the change. One example will help to explain this concept: a patient with a reversibility of 11.8% in forced expiratory volume in the 1^st^ second (FEV_1_) has the same kind of reversibility as a patient with an increase of 12.2%, despite the first being negative and the latter positive. In contrast, the latter patient (the one with an increase in FEV_1_ of 12.2%) may have a different response to treatment compared to a patient with a reversibility of 45%, despite the fact of both being positive.

Why is it so important to identify the overlap phenotype? From a clinical point of view, it is composed of COPD patients with increased reversibility and/or of smoker asthmatics with fixed airflow obstruction. Are they just the same patients with different names? Probably yes. In COPD, the positive bronchodilator response is associated with an enhanced eosinophilic inflammation in the airways in contrast to the predominance of neutrophils in irreversible COPD [12]. Similarly, the inflammation in asthma is associated with CD4+ T lymphocytes and eosinophils, and it is very responsive to corticosteroids. Conversely, neutrophilic inflammation has been linked to airflow obstruction severity both in asthma and COPD. Severe asthmatics, those with fixed airflow obstruction or those with a history of smoking, exhibit higher numbers of neutrophils in bronchoalveolar lavage fluid and biopsies [13-15]. Sputum neutrophils in stable COPD are negatively correlated with the FEV_1_ percent predicted and accelerated FEV_1_ decline, especially in patients without bronchodilator reversibility [15,16]. Neutrophilic and eosinophilic inflammations demonstrate different responses to corticosteroid treatment. These observations suggest that patients with COPD and the overlap phenotype may have some degree of eosinophilic inflammation which is more responsive to corticosteroids and has a somewhat better prognosis than patients with irreversible airway obstruction. In addition, reversible patients with COPD also present more eosinophils in sputum and higher concentrations of exhaled nitric oxide (NO) [11,15,17,18].

During exacerbations the airway inflammation becomes even more intense, with recruitment of neutrophils and eosinophils and increased CD4+ lymphocytes in the bronchial mucosa [19,20]. Studies in bronchial biopsies of exacerbated patients with mild to moderate COPD have shown a 30-fold increase in the total number of eosinophils and a 3-fold increase of neutrophils in their bronchial mucosa [21]. This increase in eosinophils during exacerbations may explain, at least in part, the clinical improvement associated with the use of corticosteroids in exacerbations of COPD [22]. Interestingly, patients with the overlap phenotype are also more likely to be frequent exacerbators (defined as the presence of two or more exacerbations per year), to have a worse quality of life, more respiratory symptoms [6,15] and to consume from 2 to 6-fold the resources used by asthma or COPD patients [23]. In addition, an increased mortality has been observed in COPD patients with peripheral eosinophilia [24]*.*

How may all these findings affect the management of our patients? It is clear that not all COPD patients have the same clinical and inflammatory characteristics and not all types of patients are represented in clinical trials. The GOLD guidelines suggest adding inhaled corticosteroids (ICs) to the treatment of patients with severe or exacerbated COPD [1]. This recommendation does not take into account the possible responsiveness to ICs. Neutrophilic inflammation present in most patients with COPD, irrespective of the number of exacerbations, does not respond to ICs [25]. On the contrary, patients with a component of eosinophilic inflammation show a significant improvement in bronchial inflammation and, more interestingly, a clinical improvement, even in mild to moderate stages of COPD [9,26,27]. In particular, one randomised trial demonstrated that prescribing ICs according to the intensity of eosinophilic inflammation in sputum was significantly superior in preventing exacerbations compared to the prescription of ICs according to guidelines [28]. This demonstrates that guidelines must be improved with recognition of personalised treatment and patterns of therapy guided by phenotypes instead of severity of airflow obstruction [29,30]. Nowadays, only the Canadian and the Japanese guidelines consider these particular characteristics of patients with COPD. The Canadian guidelines specify that: “if the asthma component (in COPD) is prominent, earlier introduction of ICs may be justified” [31]; the Japanese guideline dedicates a chapter to “Treatment of COPD complicated by asthma” [32]. The future Spanish guidelines of COPD will direct treatment according to phenotypes, and the overlap phenotype will be one of them [33].

We should not forget that COPD patients are mostly elderly, with comorbidities that require numerous drugs; therefore, avoiding the use of unnecessary medications, which may be associated with frequent and serious side effects and increased costs, must be mandatory.

Obviously, the characterization of phenotypes will require more time to be spent for the clinical evaluation of patients, but with simple investigations we should be able to diagnose the overlap phenotype in COPD [34]. Recently, by consensus a group of experts established the diagnostic criteria for the overlap phenotype. To be diagnosed with an overlap phenotype, a patient must fulfill 2 major criteria or one major and two minor among the following: A) Major criteria: very positive bronchodilator response (> 400 ml and > 15% in FEV_1_), sputum eosinophilia or previous diagnosis of asthma. B) Minor criteria: increased total serum IgE, previous history of atopy or positive bronchodilator test (> 200 mL and > 12% in FEV_1_) on at least two occasions [35]. These are quite restrictive criteria and a very conservative approach until we have more evidence from large clinical trials about characterization and differential response of the overlap phenotype. Whether you believe in the hypothesis of asthma and COPD as two expressions of the same disease (the Dutch hypothesis) or not, patients with overlap syndrome are surely different from both those with COPD and those with asthma. In many aspects they share characteristics of the two diseases, but with significant differences in clinical features such as age, lung function and cardiovascular comorbidity [36] and, even more importantly, they respond differently to existing anti-inflammatory therapies.

It is time to recognize that COPD is not a single easily treatable disease, but that it has truly different clinical, radiological and functional aspects that can mix together in distinct phenotypes, and treatment has to be tailored to the patient’s characteristics beyond simply the degree of airflow obstruction [37].
